# Tregs: Where We Are and What Comes Next?

**DOI:** 10.3389/fimmu.2017.01578

**Published:** 2017-11-24

**Authors:** Hai Zhao, Xuelian Liao, Yan Kang

**Affiliations:** ^1^Department of Critical Care Medicine, West China Hospital, Sichuan University, Chengdu, China

**Keywords:** regulatory T cells, Foxp3, regulatory innate lymphoid cells, neuropilin-1, d-mannose, amphiregulin

## Abstract

Regulatory T cells are usually recognized as a specialized subset of CD4^+^ T cells functioning in establishment and maintenance of immune tolerance. Meanwhile, there is emerging evidence that regulatory T cells (Tregs) are also present in various non-lymphoid tissues, and that they have unique phenotypes credited with activities distinct from regulatory function. Their development and function have been described in plenty of manuscripts in the past two decades. However, with the deepening of research in recent years, emerging evidence revealed some novel mechanisms about how Tregs exert their activities. First, we discuss the expanding family of regulatory lymphocytes briefly and then, try to interpret how fork-head box P3 (Foxp3), a master regulator of the regulatory pathway in the development and function of regulatory T cells, functions. Subsequently, another part of our focus is varieties of tissue Tregs. Next, we primarily discuss recent research on how Tregs work and their faceted functions in terms of soluble mediators, functional proteins, and inhibitory receptors. In particular, unless otherwise noted, the term “Treg” is used here to refer specially to the “CD4^+^CD25^+^Foxp3^+”^ regulatory cells.

## Introduction

Human beings possess a Daedalian engineering capable of eradicating both invading pathogens, from viruses to parasitic worms, and distinguishing them from the host’s own healthy tissue. Nonetheless, containment of this bloodbath is essential to preventing the host from injury due to overwhelming or misguided immune activation. Regulatory T cells (Tregs) act as the nucleus in enforcing immune tolerance (1, 2). They are mobilized as essential controller of varieties of immune responses—including allergy, autoimmunity, inflammation, and tumors immunity (3). Several present studies, additionally, have revealed a heterogeneous and multidimensional nature of tissue Tregs beyond suppressive functions. These newfound functions include helping hair follicles regeneration (4), preserving intestinal homeostasis and more importantly, participating in tissue repairing (5). Although Tregs have been reviewed extensively on all sides, most previous reviews focused on circulating subpopulation. An analysis of their novel recognized suppressive mechanisms and physical functions has not been reviewed recently.

Since their discovery in the late 1960s (6), Tregs have been extensively studied and been treated as a promising potential therapeutic tool. Determining how Tregs work is an important goal and have perplexed immunologists since they came into view. In earlier times, a variety of molecules are found to be involved in Treg-mediated suppressive activity, including cytotoxic T-lymphocyte-associated protein 4 (CTLA-4), IL-2, IL-10, TGF-β, IL-35, glucocorticoid-induced TNF receptor (GITR), lymphocyte-activation gene 3 (LAG3), granzyme B, adenosine, and cyclic AMP (cAMP). Recently, numerous studies have reported metabolic and genetic contributions, ranging from metabolic regulation estimates to mapping of immune-related genes.

**Figure 1:**
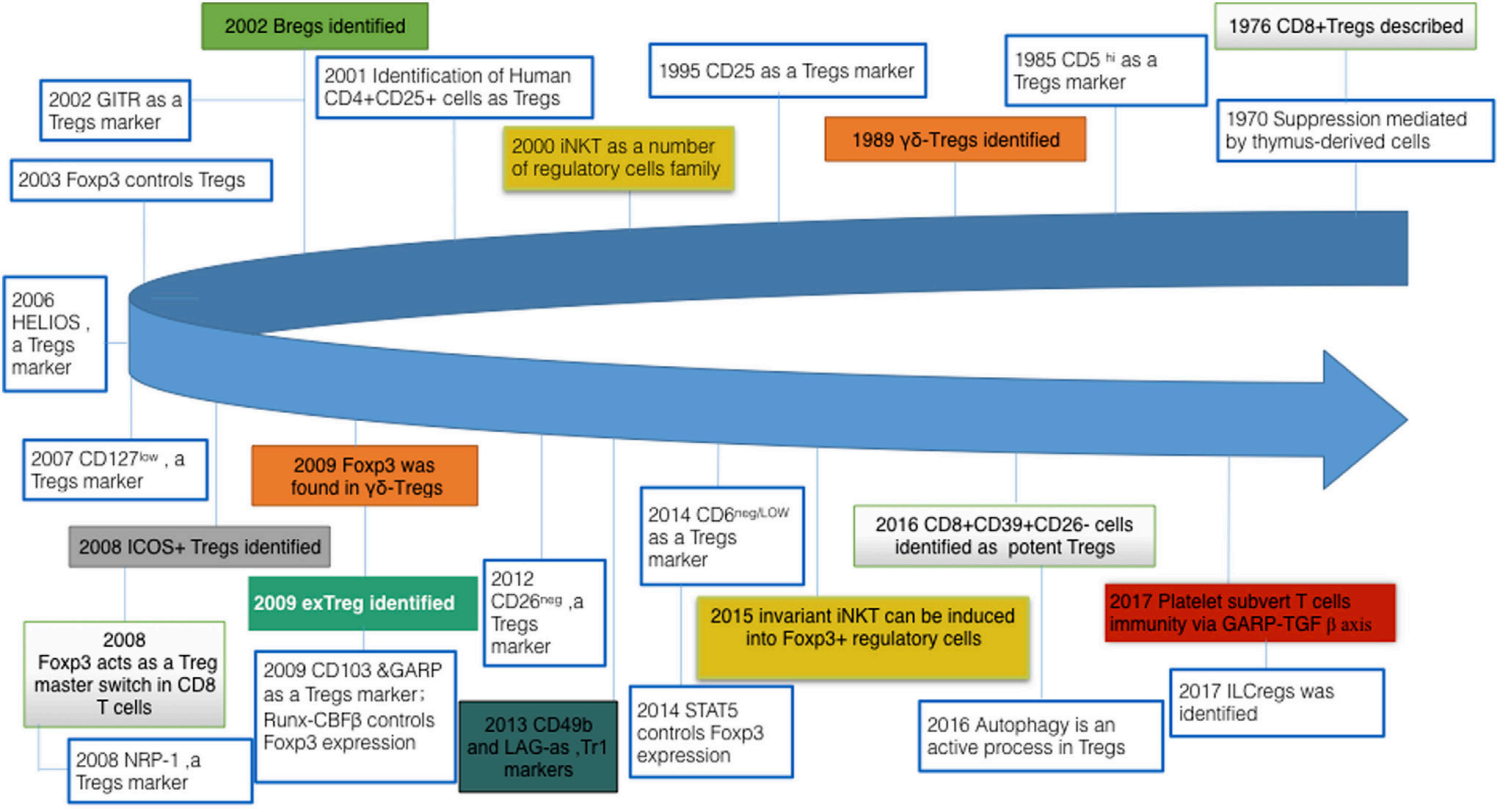
Milestone discoveries in regulatory cells field and their expanding family members. There are two crucial aspects to the “regulatory lymphocytes”: (i). A growing number of other members of regulatory cells family are gradually emerging into our sight, such as γδ-Treg and ILCregs. (ii). Meanwhile, with regard to regulatory T cells, early work was focusing on identifying their markers; present work gradually shifts to distinct functions of Tregs and their metabolomics and genomics. Tregs, regulatory T cells; Bregs, regulatory B cells; iNKT, invariant natural killer T cells; GITR, glucocorticoid-induced tumor necrosis factor receptor family-related gene; Foxp3, fork-head box P3; ICOS, inducible T cell costimulator; exTreg, T-helper (Th) 17 cells derived from Foxp3^+^ T cells without Foxp3 expression; GARP, glycoprotein A repetitions predominant; Runx-CBFβ, runt-related transcription factor–core-binding factor subunit-β complex; NRP-1, neuropilin-1; LAG, lymphocyte-activation gene; STAT, signal transducer and activator of transcription; ILCreg, regulatory innate lymphoid cells.

Remarkably, Tregs are not alone (see Figure 1). At first, three main types of CD4^+^ regulatory cells have been firmly established: IL-10-producing Tr1 (type 1 regulatory T) cells, TGF-β-producing CD4^+^ Th3 (T helper 3) cells, CD4^+^CD25^+^Foxp3^+^T cells. Both Tr1 and Th3 lack fork-head box P3 (Foxp3) expression and several of cytokines were shown to account for their inhibition. Besides, the population of immunosuppressive cells also cover so-called myeloid-derived suppressor cells (MDSCs), regulatory B cells (Bregs), regulatory γδ T cell (γδ-Τregs), immunosuppressive plasmocytes (ISPC), etc. Just very recently, a regulatory subset of ILCs (innate lymphoid cells) has also been identified (7).

Among these regulatory cells, CD4^+^CD25^+^Foxp3^+^ regulatory T cells are the most physiologically relevant due to their broad and indispensable roles. Hence, we focus on Tregs in this review and we should keep in mind that all regulatory cell members do not act in isolation but rather have myriad connections with each other to accomplish this biological play altogether. So we would not introduce heretofore the cutting edge pertinent to different phenotypes of regulatory cell members in detail. Herein we will introduce some recent bright research about Tregs suppressive mechanisms and try to explore their possible molecule targets.

It has been universally accepted that the physiological function of Treg is essential for the restraint of fatal autoimmune and inflammatory responses. There remains significant room and it is still necessary to identify unrecognized pathways regulating Treg development and function. Similarly, we should also keep in mind that relevant research cannot be considered independently. Rather, they interact to make up an elaborate, sophisticated manipulation of the immune system.

## Foxp3 and Tregs

The transcription factor Foxp3 is critically important for the development and function of Tregs (8). Foxp3 not only can keep the cells on right developmental tracks toward a suppressive phenotype, but also seems to be a prerequisite to for stabilizing the Treg lineage (9). Furthermore, loss of Foxp3 expression over time impairs the suppressive activity of Tregs (10, 11).

The genomic region of the *Foxp3* locus has several conserved noncoding sequences, designated as CNS 0–3. Each sequence gets involved in different signaling pathways respectively. CNS0 represents a role in initiating Treg-SE (specific super-enhancers) activation to induce Foxp3 expression (12). Present findings also revealed that myeloid/lymphoid or mixed-lineage leukemia 4-AT-rich sequence-binding protein-1-CNS0 region complex promotes looping of *Foxp3* promoter (13). CNS1 contains binding sites for the nuclear factor of activated T cells and the activator protein 1, which is indispensable to TGF-β signaling mechanisms (14). Additionally, CNS1 is critical for TGF-β-induced Foxp3 induction in peripheral CD4+ T cells but not in thymocytes (15). CNS2, activated by TCR expression and interleukin-2, has varieties of transcription factor binding sites such as cyclic adenosine monophosphate response element-binding protein, signal transducer and activator of transcription (STAT5), and runt-related transcription factor (RUNX) (16, 17). Specially, RUNX1–CBFβ complex β binding to CNS2 is crucial for sustaining a high and stable level of Foxp3 expression in Treg cells (18). Last in sequence but not least in importance, CNS3 acts like an essential element for Foxp3 induction during thymic and peripheral Treg differentiation by recruiting c-Rel and other transcription factors (19). Summarize, expression of Foxp3 alone is not sufficient for conferring and maintaining Treg cell function and phenotype. Foxp3 can be regarded as one of large transcriptional complexes, which contain some other transcriptional factors. For the interior, subunits of Foxp3 can bind different transcriptional factor to achieve development and functions of Treg.

Though Foxp3 acts as a master regulator of the suppressive pathway in the development and function of Treg, Foxp3 is not necessary for survival of Treg precursors (9). In addition, Foxp3 is not just for Tregs alone since it can be expressed in activated non-suppressive CD4^+^CD25^−^ Tregs (20). More specifically, T cells posing the Treg-cell specific epigenetic changes are not completely overlap with those expressing Foxp3. To fully understand the relationship among CD25, Foxp3 and Treg epigenome, we refer readers to a comprehensive review on identity of Treg in Ref. (21).

## Tissue Tregs

It is becoming increasingly appreciated that Tregs accumulated in various nonlymphoid tissues are important parts of immune system. Tissue Tregs have unique phenotypes, different TCR repertoires, distinct functions. Identification of nonlymphoid tissues Foxp3^+^CD4^+^ regulatory T cells fuels the notion that human immune system possesses a second critical function: maintaining organismal homeostasis. In this part, we will discuss tissue Tregs in four parts of the body—visceral adipose tissue (VAT), skeletal muscle, mucosal interface and hair follicle.

Regulatory T cell in VAT (VAT-Tregs, also known as “Fat Tregs”), as one of the well-characterized examples of tissue Tregs, seem to retain more strong suppressive ability since transcriptional level of IL-10 is 10^2^-fold higher than that in lymph nodes (22). Meanwhile, VAT-Tregs display elevated CCR1, CCR2, CCR9, CXCL10 and low CXCR3 expression which is induced by PPARγ (peroxisome proliferator-activated receptor-γ) (23). They are abundant in VAT of lean mice, and instead, they would dramatically decreased in insulin-resistant animal models of obesity (22). Their development was largely due to effects of PPARγ, which is a master regulator of the accumulation, phenotypes and functions of adipose tissue Treg cells (23). Additionally, the transcriptional regulator basic leucine zipper transcriptional factor ATF-like, interferon regulatory factor 4, together with IL-33 and its receptor, suppression of tumorigenicity 2 (ST2; also known as IL-1RL1) play an indispensable role in VAT-Tregs differentiation (24). VAT-Tregs have been suggested to get involved in some metabolic disorders such as atherosclerosis (25), obesity (26), and diabetes (27), strengthening this type of tissue Tregs as a promising target for therapeutic interventions.

Muscle-resident Tregs was first identified in genetically dystrophic mice in 2013 (28). They display enhanced expression of IL-10, Granzyme B, plate-derived growth factor, amphiregulin, CCR1, CCR2 and of particular importance is ST2 (IL-33 receptor). In addition, muscle-resident Tregs are supposed to be exported from the thymus since they are accompanied with high levels of Helios and Neuropilin (28). In the synergic sequence of events underlying muscle repair, muscle-resident Tregs adequately contribute to this process and come into the limelight. A more recent research has revealed the close correction between muscle-resident Tregs and muscle recovery from injury (29). The authors first identified that muscle-resident Tregs were reduced in aged mice who were characterized by delayed or impaired muscle recovery. Then they found administration of IL-33 restored the Treg population and enhanced regeneration, opening a new therapeutic avenue for poor wound healing to explore. However, the role of amphiregulin, which is considered to directly modulate muscle homeostasis and regeneration (5, 28), in the research seems unclear. Moreover, how muscle-resident Tregs capture damage signal and how they subsequently export from the thymus awaits elucidation.

A large fraction of Tregs accumulate at mucosal interfaces, especially the lamina propria of colon. Intestinal microenvironment provides the venue where commensal microbes accrete with immune cells. Therefore, the TCR repertoire of colonic Tregs is distinct from that of colonic effector T cells or Tregs in other tissue sites. Intestinal Tregs exhibit increased expression of IL-10 and TGF-β, which is in accordance with the unique array of antigens they are exposed to (30). Just like muscle-resident Tregs, more than half of the intestinal Tregs display ST2. IL-33 not only facilitates intestinal Tregs differentiation but also promotes their accumulation in inflamed tissues (31). Additionally, a significant proportion of intestinal Tregs coexpress the transcription factor GATA-binding protein 3 (GATA3) which regulates ST2 expression (32, 33). Herein, we did like to spend a small amount of space to compare intestinal Tregs with ILCregs. ILCregs, namely regulatory innate lymphoid cells, were found to exist in mouse and human intestines (7). In stark contrast with intestinal Tregs and other innate lymphoid cells, ILCregs lack typical transcription factors such as Foxp3, GATA3, and retinoic acid receptor-related orphan receptor-γt. They suppress the functions of innate lymphoid type 1 cells and innate lymphoid type 2 cells *via* IL-10 and TGF-β mainly. Though exploration of gut-associated regulatory cells in humans is only beginning, the interactions between immune system and them promise to be particularly fruitful areas of future study.

The presence of tissue Tregs in diverse nonlymphoid organs in both mice and humans has attracted a great deal of attention over the past few years. VAT-, skeletal muscle-, and gut-associated tissue Tregs are at the cutting edge in this field. However, it is also important to deepen our understanding of Tregs in several other regions, including central nervous system, hair follicle, and cardiac muscle, especially their specialized roles of each in regulating local immune responses and their tissue-specific functions.

Given that Tregs are potent mediators of the immune response for the maintenance of dominant self tolerance and immune homeostasis, there is considerable interest in determining their mechanisms of action. The mechanism by which Tregs exert their function has been pursued relentlessly for decades. It has become apparent that Tregs have evolved a wide range of mechanisms by which to maintain its role in immune responses. These mechanisms can be classified into categories of: (i) suppressive cytokines (IL-10, TGF-β, IL-35, etc.); (ii) metabolic disruption (cAMP, CD39, CD73, etc.); (iii) modulation of antigen presenting cells maturation or function; and (iv) suppression by cytolysis. But are there more undiscovered mechanisms and/or molecules that mediate Tregs function? It is a matter of debate in the field and we should be aware that it has no end in the near future.

## Soluble Mediators Derived from Treg

### Cyclic AMP

Treg contains a high concentration of cAMP, while effector T cell has non-detectable cAMP loads (34). But no consensus has been reached as to which exquisite mechanism are closely related to cAMP. There are presently two different theories. In one scenario, CD39 and CD73, cell surface ectoenzymes that convert ATP/ADP to adenosine in synergy, are highly expressed on Treg (35). As a result, high quantities of adenosine are released into the extracellular space. Then this small molecule activates A2a receptors on effector T cells and increases intracellular cAMP to inhibit TCR-mediated signaling by preventing zeta-chain-associated protein kinase 70 (ZAP70) phosphorylation and activator protein-1 activation (36). The subsequently decreased TCR signaling leads to impaired CD25 expression and IL-2 production which are detrimental to effector T cells proliferation and activation.

Besides, Tregs themselves carry innumerable cAMP and can directly delivery this small molecule to effector T cells *via* gap junction to exert suppressive function as mentioned. This communication type was demonstrated by inserting calcein which is the unique permeable dye for gap junction (37, 38). There are two points worthy of our attention.

**Figure 2:**
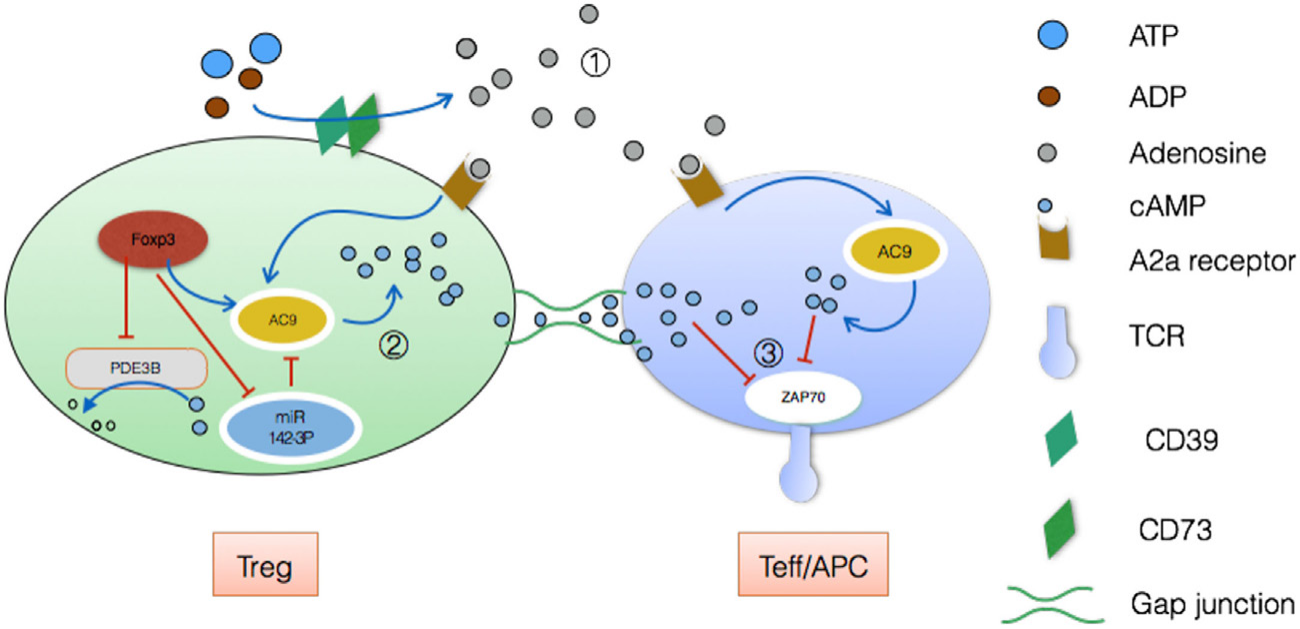
Treg-mediated suppressive function *via* cyclic AMP (cAMP). ① Through expression of the ectoenzymes CD39 and CD73, Treg drives the accumulation of adenosine extracellularly, which disrupts Teff metabolism, leading to anergy. During this process, adenosine activities high-affinity A2a receptors, with a result of plenty of cAMP by means of adenylyl cyclase 9 (42). ② On the other hand, cAMP pool in Treg can be poured into Teff/APC by means of GJIC (43). Connexin43 plays an irreplaceable role in this delivery (44). ③ Finally, accumulative cAMP inhibits TCR-mediated signaling by preventing zeta-chain-associated protein kinase 70 (ZAP70) phosphorylation. This decreased TCR signaling leads to impaired Teff/APC activation and proliferation eventually (45).

First, Foxp3 can strongly downregulate *Pde3b* locus while phosphor-diesterase3b hydrolyzes cAMP and cGMP (9). Second, Foxp3 additionally suppresses expression of miR-142-3p which is a potent inhibitor of adenylyl cyclase 9 (AC9) while AC9 retains the ability to generate cAMP (see Figure 2) (39). Consequently, Tregs contain comparatively high levels of AC9 and cAMP, leading to the suppression of effector T cells and antigen-presenting cell (APC) after delivering cAMP *via* gap functional intercellular communication (GJIC). To be specific, GJIC has been emphasized as a novel pathway for Treg-mediated suppression. Kuczma et al. showed that deviant expression of connexin 43 (an important element of gap junction) impairs suppressive function of Tregs evidently (40). Further analyses revealed that both connexin43 and its analog alpha-connexin carboxyl-terminal peptide 1 can enhance gap junction aggregation (41). Hence, we have adequate reason to believe that all these involved will show promising application to regulating immune homeostasis.

### TGF-β

TGF-β, namely transforming growth factor-β, is usually regarded as one inhibitory cytokine. Th9, Th17, and Tregs all require this cytokine for their development though they still need additional cytokine signals for their eventual fates, respectively (46). After activation *via* TCR, Foxp3^+^ T cells express glycoprotein A repetitions predominant (GARP) which increases latent TGF-β activation. GARP^−/−^ Tregs can still secrete latent TGF-β which does not have biologic activity. The source of activated TGF-β was largely the GARP/latent TGF-β complex instead of only latent TGF-β. Notably, Helios, but not FoxP3, dominates the expression of the GARP/latent TGF-β on activated human Treg (47). Considering its important role in activating latent TGF-β, GARP may represent an additional target to inhibit Treg suppression in cancer or augment suppression in autoimmunity (48).

d-mannose, a C-2 epimer of glucose, widely exists in a free state in some plant peel such as citrus skin. According to the latest knowledge, d-mannose can induce naive T cells differentiation toward Tregs in a dose-dependent manner (0–50 mM) by promoting TGF-β activation (49). After oral administration of d-mannose into models of autoimmune diabetes and airway inflammation, the researchers found immune responses of the objects showed an immunoregulatory phenotype and subsequent tolerance. Instead, inhibition of TGF-β signaling can counterbalance the d-mannose-induced Treg generation. More importantly, long-term supplementation with d-mannose would produce no adverse consequence. Combined with its easy acquisition, d-mannose-involved immune responses have particular implications for a similar clinical therapy for some frequently occurring diseases such as type 1 diabetes in humans.

As mentioned before, Tregs exhibit functional and phenotypic heterogeneity. Konkel et al. showed that TGF-β signals limit Treg suppression of Th1-responses but are key for Treg function in the colon (50). They revealed a series of previously unrecognized role of TGF-β, including maintaining CD103 expression, boosting G-protein coupled receptor 15 (a colon-specific trafficking molecule) expression and inhibiting GPR174 (a G-protein-coupled receptor for lyso-phosphatidylserine) expression. The study has dramatically broadened our understanding of TGF-β in immune responses.

**Figure 3:**
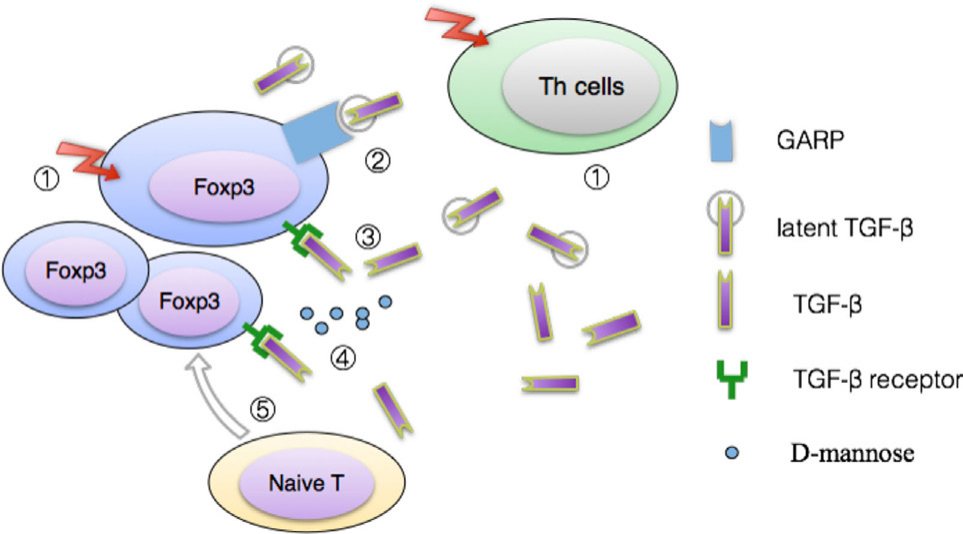
Model for TGF-β production by Treg. ① The latent form of TGF-β can be released from both activated Tregs and T helper cells (53, 54). Inside latent TGF-β form, latency-associated peptide (LAP) is bound tightly to active TGF-β, acting as a shield which separate active TGF-β from its receptor. ② Glycoprotein A repetitions predominant protein (GARP), a kind of transmembrane anchor to keep latent TGF-β cling to the cell surface, is a cell surface receptor on activated Tregs, platelets, but negligible expressed on Th clones (55, 56). Combined with LAP and mature TGF-β, GARP represents the third part of a muti-protein complex in activated Tregs. Or, GARP can be regarded as a covalent receptor for latent TGF-β in active Tregs. Subsequently, association of TGF-β with GARP induces activation of the latent complex *via* integrin α_v_β_6_ or integrin α_v_β_8_ (57). ③ Active TGF-β interacts with its specific receptors to induce cellular responses. ④ Recent research disclosed that d-mannosem can upregulate levels of integrin a_v_b_8_ and reactive oxygen species generated by increased fatty acid oxidation, which facilitates activation of the latent TGF-β (49). ⑤ In synergy with IL-2, TGF-β promotes the conversion of naive CD4+ T cells toward Tregs by upregulating expression of Foxp3 (58, 59).

Summarily, some early *in vitro* studies indicated TGF-β was not essential prerequisite for the function of naturally occurring Tregs (51) but ensuing research gradually overturned this antecedent conclusion. Especially, relationships between TGF-β and IL-2, GARP, Gfi-1(growth factor independent 1) still need further investigations (see Figure 3). For a more comprehensive description of how TGF-β participating in Tregs-mediated suppression, especially in the interplay between immune cells and the microbiota in the gastrointestinal tract, we recommend a recent review (52).

### Neuropilin-1

Expression of Neuropilin-1 on human Tregs has been a contentious subject (60, 61). In the past few years, awareness of Neuropilin-1, known as a coreceptor for both semaphorin family members and vascular endothelial growth factor, has grown, as has interest in its potential therapeutic role in promoting antitumor immunity. In contrast to the silent expression of healthy donor peripheral Tregs, Neuropilin-1 is expressed by approximately 90% of tumor infiltrating Tregs in cancer patients (62) and increased percentage of human Neuropilin-1^+^ intratumoral Tregs correlates with poor prognosis. Being here, we highlight a recent study by Overacre-Delgoffe et al. who proved the key role of Neuropilin-1 in Treg function fragility (63). They found Treg-restricted deletion of Neuropilin-1 facilitates its IFN-γ production, which extends previous data that a small subsets of Tregs can generate IFN-γ (64). Very importantly, *Nrp1* deficiency in Tregs would not induce any autoimmune or inflammatory disease of host, indicating that Neuropilin-1 is dispensable for prevention of autoimmunity or maintenance of immune homeostasis.

Regulatory T cells, as a major barrier to effective antitumor immunity, have multifaceted roles in promoting tumor development through immune escape and angiogenesis. Though it is not necessary for immune homeostasis, Neuropilin-1 is yet indispensable for Tregs to limit antitumor responses (65). In the tumor context, Treg-restricted Neuropilin-1 deletion can revert antitumor responses based on more than one pathways: first, Neuropilin-1 directly enforces Treg stability and function in the tumor microenvironment. Neuropilin-1 ablation significantly impairs stability and suppressive activity of Tregs *via* inhibiting Akt functions. Second, Neuropilin-1-deficient Tregs show proinflammatory phenotype—secreting IFN-γ—instead. IFNγ is an important activator of macrophages and inducer of MHC II molecule expression. It can limit Treg expansion (66) and drives Treg fragility to promote antitumor immunity. Recently, it was observed that IFN-γ helps to prevent relapse by keeping the tumor in an ischemic state (67). Finally, the authors demonstrated that Neuropilin-1-deficient Tregs also negatively impact the function of surrounding intratumoral normal Tregs.

This milestone discovery makes Neuropilin-1 one potential therapeutic target, which could restrain Treg-mediated antitumor effects without inducing autoimmunity. As high-level Tregs in intratumoral setting are key players in tumor escaping and angiogenesis, selectively targeting intratumoral Tregs while maintaining peripheral tolerance is of vital significance. If we can turn the powerful foe into mighty friend, Treg will become one advanced weapon against cancer and other diseases.

### Amphiregulin

As an activating ligand of the epidermal growth factor receptor, amphiregulin is expressed by multiple cell types in a variety of inflammatory setting including group 2 innate lymphoid cells, basophils, mast cells and Tregs. A previously unrecognized population of Tregs with amphiregulin-expressing was found accumulating in injured skeletal muscle (28) and inflamed colon of mice (31). Recently, Arpaia et al. have showed that amphiregulin-deficient in tissue Tregs induces severe tissue damage but without impaired suppressive function (5). They demonstrated that Tregs have a major direct role in tissue repair which is invoked by separable cues. For years, as we know, congenital deficiency in Tregs causes fatal autoimmunity in so called *scurfy* mice (68), and IPEX syndrome in humans (69). The etiology of these disorders has mostly been attributed to the failure of Tregs to exert suppressive function. However, this conclusion needs to be fresh and up-to-date, with the recognition of Tregs’ non-redundant role in tissue repair. Besides, these amphiregulin-expressing Tregs also display a characteristic gene expression and a specific TCR repertoire in stark contrast with that of splenic Tregs. Amphiregulin production in tissue Tregs is elicited *via* IL-33 and IL-18 rather than TCR signaling. That amphiregulin expression is dispensable for their suppressive function reinforces the heterogeneity of Treg compartments. Heterogeneous subpopulations of Tregs are possibly armed with diverse functions, and Tregs are not merely immune component.

Although amphiregulin is dispensable for Treg suppressor function, it does not detract amphiregulin from being a promising clinical biomarker and therapeutic target. For example, increased amphiregulin levels have been found in non-neoplastic diseases, including inflammatory diseases (70, 71) and autoimmune diseases (72, 73). Furthermore, amphiregulin is involved in cancer progression and has become the focus of several basic, translational, and clinical investigations (74–76). A growing number of studies support the concept that amphiregulin is indispensable for tissue integrity, and ultimately to abstain tumor development. In this regard, to better understand its upstream regulation and its interaction with other signaling molecules still need to be demonstrated. Admittedly, the mechanisms regulating amphiregulin expression, the relationship between amphiregulin and IL-33, and the exact role of amphiregulin in heterogeneous Treg subpopulations remains elusive. Targeting specifically the crosstalk between immune and epithelial cell *via* amphiregulin still holds promise for the development of therapeutics to combat non-neoplastic diseases and cancer.

### Interleukin-34

Interleukin-34, a newcomer of human interleukin family, first described in 2008, was found to be crucial in cell proliferation, differentiation, inflammation, angiogenesis, migration and adhesion (77). It has another two distinct receptors, namely PTP-ζ (the receptor-type protein-tyrosine phosphatase zeta) and CD138 in addition to the earlier demonstrated colony-stimulating factor-1 receptor (CSF-1R). The affinity of IL-34-CSF-1R binding is higher than CSF-1-CSF-1R binding since IL-34 recruits two domains of CSF-1R, while CSF-1 recruits only one (78).

Bézie et al. proved IL-34 retains immunosuppressive properties and they identified IL-34 as a tolerogenic cytokine with miscellaneous physiological functions (79). They first observed that IL-34 is specifically expressed by CD4^+^CD45RC^lo^ Foxp3^+^ and CD8^+^CD45RC^lo^ Foxp3^+^ Tregs. In more detail, nearly half of the CD3^+^ Foxp3^+^ Tregs express IL-34, indicating its specific role in Treg function. Still other studies found diverse functions of IL-34 such as inducing proinflammatory cytokines (80), driving regulatory macrophages generation (81).

Interleukin-34 is involved in the development of a series of diseases such as rheumatoid arthritis (82, 83), inflammatory bowel disease (84) or neoplastic disorders (85, 86). The biology and underlying mechanism of this interleukin remain debatable today and its relation with Tregs still awaits further elucidated. What we have known is far from enough, but we still have faith in its potential key role in immune regulation.

### Interleukin-35

Interleukin-35, a newly discovered member of IL-12 family, is the most important cytokine with anti-inflammatory properties besides TGF-β and IL-10. IL-12 family, including IL-12, IL-23, IL-27, and IL-35, are composed by two of the following five subunits—p19, p28, p35, p40, and Epstein–Barr virus-induced gene 3. To be specific, two chains forming the heterodimeric IL-35 are the α-chain (p35, shared with IL-12) and β-chain (also a component of IL-27), both of which are highly expressed by Tregs other than effector cells or APCs (87).

**Figure 4:**
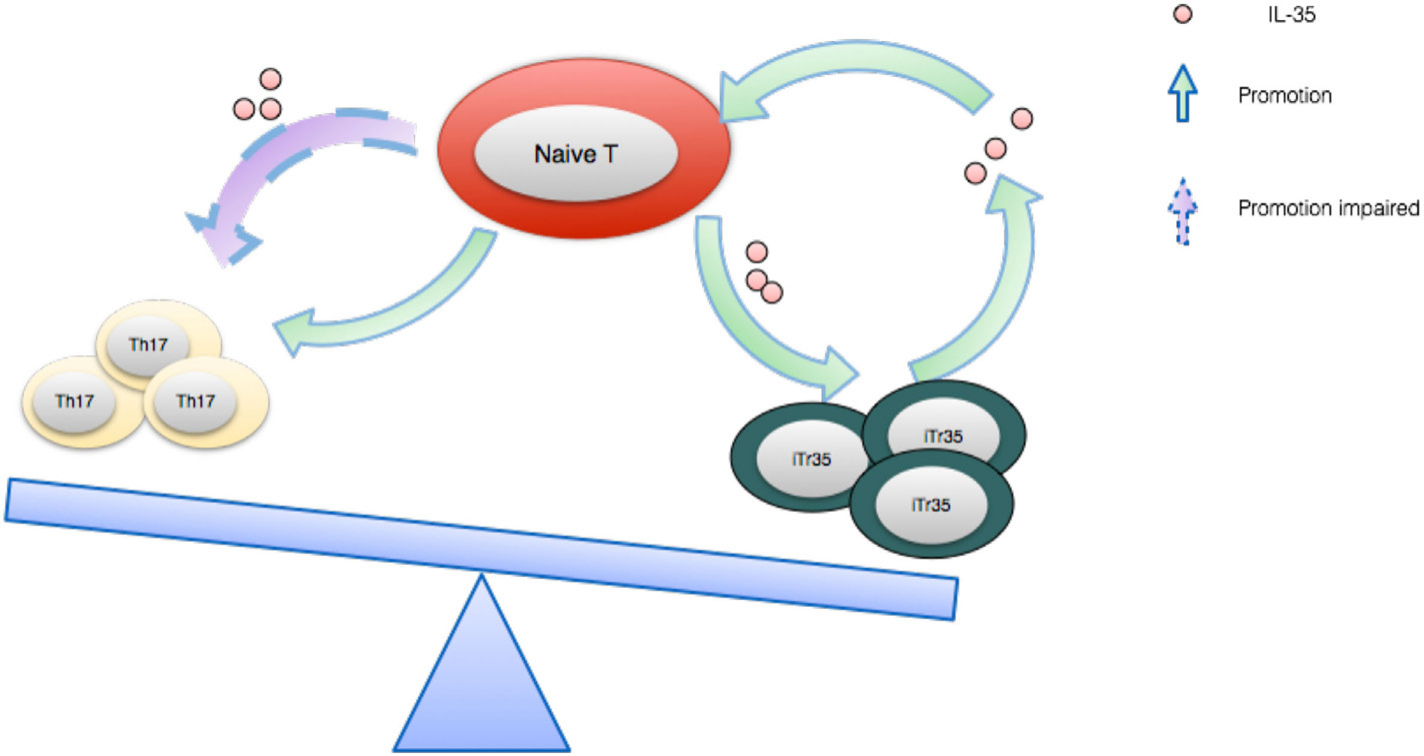
The IL-35 expression feedback loop and how it balances Th17 cells and iTr35. IL-35 induces naive T cells to differentiate toward regulatory iTr35 cells while the latter further secrete higher concentration of IL-35, forming a positive feedback cycle. Conversely, IL-35 generates a negative effect on the differentiation and function of Th17 cells.

While IL-12, IL-23, and IL-27 are proinflammatory immune cytokines, IL-35 is a purely immunosuppressive cytokine. For the moment at least, Tregs are thought to be the main source of IL-35 and this potent cytokine is indispensable to their maximal suppressive capacity (88). Of note, not only is IL-35 in a position to directly suppress effector T cell response, it is also able to induce iTr35 cells generation (89). After being secreted by Tregs, IL-35 subsequently acts on its target cells by binding to its receptor, which is composed of IL-12β2 and gp130. Once combined, the signal will be transducted through STAT1 and STAT4, which eventually results in a feedback loop promoting IL-35 expression (87) (see Figure 4). In addition, IL-35 has been well demonstrated to enhance the proliferation of nTregs as opposed to IL-12 and IL-27(90).

Recently, emerging studies of IL-35 involvement in human diseases have been reported, including inflammatory disease, autoimmune diseases, neoplastic disease. In particular, Turnis et al. have shown that Tregs-derived IL-35 is enriched in tumors and promotes exhaustion of effector T cells in this microenvironment (91). They speculated that IL-35 becomes a more prominently utilized suppressive mechanism in cancer especially. There is no doubt that IL-35 exerts an important role in the pathogenesis and development of multiple disease although its accurate role remains somewhat controversial. Indeed, studies focusing on IL-35 have gradually changed from animal models into human studies, suggesting a promising therapeutic target of IL-35. The primary challenge we face is the difficulty to produce IL-35. How can similar IL-12 family members produce diverse functions? How do these subunits exactly work? If we pair p35 with p28 or p19, will the compound present proinflammatory or anti-inflammatory function? Furthermore, as a newly recognized inhibitory molecule, what we have known may be just a tip of the iceberg. In addition to its advantageous effects, we should meanwhile take potential deleterious impairment on board.

It is worth mentioning that the suppressive activity of IL-35 is not confined to CD4^+^ Tregs. A subpopulation of CD8^+^ Tregs was also identified to inhibit the proliferation of effector T cells in a similar IL-35-dependent manner (92).

## Effects of Ectoenzymes and Inhibitory Receptors on Treg Suppression

### CD39 and CD73

CD39 and CD73 play strategic roles in transformation from ATP-induced proinflammatory mileu to adenosine-induced anti-inflammatory mileu. About 80% of Foxp3^+^ Tregs retain a high concentration of these two ectonucleotidases (93). And more, Tregs also secrete exosomes containing CD39 and CD73 and these exosomes have been found to suppress effector T cells proliferation (94).

Adenosine generated by CD39 and CD73 on Tregs can bind the A_2A_ adenosine receptors on effector T cells and enhance intracellular cAMP levels to suppress their function. Hence, they have been increasingly used as functional markers of Tregs. Notably, CD39 is regarded as the rate-limiting component of this ectoenzymatic chain. Effects of them upon adenosinergic loops comprise a component of the suppressive machinery of Tregs. One recent review by Cekic and Linden (93) has elaborated this labyrinth among them and again we will not reiterate them here. Herein we highlight some recent advance in understanding of CD39 and CD73.

Indeed, there exists a substantial obstacle to the understanding of immune regulation, largely because the major self-antigens recognized by Tregs have far remained elusive. In addition to being an ATP hydrolase, Gruenbacher et al. recently proved CD39 on Vγ9Vδ2 T cells dephosphorylates and thus desensitizing phosphoantigens-associated responses (95). With the boom of chimeric antigen receptor T cell therapy, identification of Treg-associated antigen appears to be particularly important. Hydrolyzing specific antigens would be un previous unrecognized pathway Tregs functions, at least for Vγ9Vδ2 T cells. Recently, Leonard et al. also identified two Treg clones recognized distinct non-overlapping MHC-class-II-restricted peptides derived from transient receptor potential cation channel subfamily M member 8-channel-associated factor 3 (96). These bright works could be exploited as a potent strategy for identifying the Tregs antigen relevant to human autoimmunity.

Notably, in contrast to murine Tregs, CD73 is predominantly expressed intracellularly in human Tregs. It has been reported that in humans, only 1%-5% of circulating FOXP3^+^CD4^+^ T cells expresses CD73 while its surface expression on human Tregs can be induced upon activation (97). Also, the role of CD73 on Tregs is confirmed since inhibition of it impairs their suppressive capacity. Indeed, besides its enzymatic function, CD73 can also be regarded as one adhesive molecule that can regulate cell interaction with extracellular matrix components to mediate cancer invasive and metastatic properties. Several studies revealing the relationship between CD73 and clinical prognosis have sprung up (98–103). Further, anti-CD73 therapy alone (104) or combined with other inhibitory molecules (105) have produced inspiring benefits in preclinical models. Admittedly, these advancements may reflect the relationship between CD73 and tumor microenvironment rather than Tregs alone. But they are still instructive for us to study Tregs in depth. With the advent of Generation 3 immunoncology, molecule target ranks enormous position and the future of CD73-targeted therapy holds even brilliant prospects.

### CTLA-4

Cytotoxic T-lymphocyte–associated antigen 4 (CD152) is regarded as a “leader” checkpoint inhibitor since it terminates potentially autoreactive T cells at earlier stage in contrast to another star—programmed cell death 1 (PD-1). The CTLA-4 level of conventional T cells will rise once they are activated yet it is constitutively and especially expressed on Tregs in the resting immune system. It is well demonstrated that CTLA-4 gets involved in crucial function of Tregs given that CTLA-4 binds B7 with a higher affinity than CD28 (106). This competition for ligand binding sets up a potent immuno-modulation target—CTLA-4 blockade induced an increased availability of ligands for CD28 binding and the reverse is also true. Competition with CD28 on T cells for B7 signaling, negatively regulating APCs *via* B7 (107) and Trans-endocytosis of B7 (108) constitute the basic biology of CTLA-4. Many molecules get involved in CTLA-4-mediated downstream signaling pathway in T cells, including CD3ζ chains (109), ZAP-70 (110), phosphoinositide 3-kinase (111), AKT (protein kinase B) (112) and protein kinase C isoform (113), *etc*. However, none of these theories has won a landslide victory and the debate over this molecule will last in the near future.

Since *Ctla4* is a target gene of Foxp3, this leads to the concept that CTLA-4 might engage in directing the homeostasis and function of Tregs. Firstly, CTLA-4 in Tregs can act as an intrinsic brake on their proliferation. Deletion of CTLA-4 resulted in enhanced Tregs multiplication (114). Further, injecting mice with a CTLA-4 specific blocking antibody also rapidly induce Tregs proliferation (115), fueling the original observation. By comparison, loss of IL-10 expression of Tregs fails to induce systemic autoimmunity, indicating that systemic immune homeostasis has tight association with the expression of CTLA-4 rather than IL-10 (116). In contrast, blockade of B7 molecules with CTLA-4-Ig remarkably decreases the number of memory Tregs which exhibit stronger suppressive function (117). This manipulation, however, induces negligible effect on the naive Treg subpopulation (117). Summarily, loss of CTLA-4 increases Tregs numbers while loss of CD28 results in markedly decreased Tregs numbers. Second, CTLA-4 plays a key role in Tregs function, at least in some settings. Loss of CTLA-4 in Tregs can lead to impaired suppressive activity of Tregs (118) and aberrant function of conventional T cells (119). Recent data have proved CTLA-4 expressed by Tregs can prevent inflammatory tissue attack in arthritis context (120). Very low dose of IL-2 (less than 5 IU/ml) was also found to enhance Tregs function in a CTLA-4-dependent manner (121). It is archived *via* selective phosphorylation of STAT-5 in Tregs rather than other cell lineages. But there appeared a different voice meanwhile—blocking the function of Treg with CTLA-4 antibodies *in vitro* fail to produce impaired Tregs suppression (122). This embarrassment was mainly due to CTLA-4 sharing communal ligands with B7. The ultimate way-out to this solution maybe lies in ideally interrupting CTLA-4 pathway while leaving CD28 pathway intact.

In a surprising twist, controversies over CTLA-4 of Tregs do not affect their clinical application. It is an emerging diagnostic marker and therapeutic target for human diseases at present. For example, in early onset breast cancer, immunohistochemistry expression of CTLA-4 was investigated and its mean percentage value in intratumoral lymphocytes reached 8.24% (123). There is also a significant correlation between CTLA-4 expression and overall survival in different cancer cases (124). More importantly, CTLA-4 has been extensively studied for immunotherapy besides PD-1. Monoclonal antibodies targeting CTLA-4 (e.g., ipilimumab, tremelimumab) have emerged as potent weapons against cancer and show great promise in treating a broad range of diverse tumor types (125, 126). Anti-PD-1 and anti-CTLA-4 have complementary activities and the combination of PD-1 and CTLA-4 blockades brought about more benefits than either agent alone (127, 128).

Considering the sustained expression on all Tregs, it seems tenable to consider CTLA-4 as a core suppressive molecule. This hypothesis is fueled especially in the burgeoning trend of low dose IL-2 therapy. As discussed previously, CTLA-4-mediated suppression is a significant pathway by which low dose IL-2 confers enhanced suppressive potential to Tregs (121). We can speculate whether CTLA-4 is the priority option and whether CTLA-4 alone is enough for Tregs function. Admittedly, a more detailed framework is still required in order to clarify this controversial but non-negligible player.

### PD-1

The coinhibitory receptor PD-1 (also named CD279) was discovered in 1992 (129) and is mainly expressed on activated CD4^+^ T cells and CD8^+^ T cells as well as on B cells in the periphery. Just like CTLA-4, as a member of CD28 family, PD-1 delivers a negative signal when interacting with its ligands (i.e., PD-L1 and PDL2). PD-1 has been an paramount target of immunotherapy now used in the clinic, though a large subset of patients fail to respond to anti PD-1 immunotherapy. The relationship between PD-1 and Tregs is just kicking off. Woods et al. found that NED (patients with no evidence of disease) displayed increased percentages of Tregs postnivolumab (anti-PD1, Bristol Myers Squibb) while relapsing patients did not (130). They concluded PD-1 blockade increased pSTAT3 (phospho-signal transducer and activator of transcription 3) expression and subsequently enchanced Tregs percentages *via* enhancing IL-10 production (130). Another recent study by Ha et al. also proved PD-1^+^Tregs are characterized with stronger suppression function during chronic viral infection (131). In synergy with TGF-β, PD-1 downregulates the threshold of TGF-β-mediated signals and thereby induces the cytodifferentiation of naive T cells toward iTreg cells (132). Then, PD-L1 restrains effector T cell responses by enhancing the proportion of iTregs (132). Furthermore, a recent study by Overacre-Delgoffe et al. have revealed another underlying relationship between PD-1 and Tregs. They demonstrated IFN-γ-induced Treg fragility is required for an effective response to PD-1-targeted immunotherapy (63). This study partly answers the key issue why some patients are featured with mute responsiveness to PD-1 blockade.

### TIGIT, LAG-3, Tim-3

Only a few subsets of cancer patients benefit from anti-PD-1 and anti-CTLA-4 therapy and these targeted medicines may induce unavoidable side effects. This imperfection has leaded deep interest in targeting of other immune checkpoint receptors.

T-cell immunoglobulin (Tim) and immunoreceptor tyrosine-based inhibitory motif domain (also termed as WUCAM, Vstm3, VSIG9), a recently defined new immune checkpoint, was first identified as another member of CD28 family. Its expression is limited to lymphocytes, especially high-level expression on regulatory T cells and NK cells (133). TIGIT binds two ligands, CD112 and CD155, which are expressed on APCs, T cells, and tumor cells, *etc*. To test how TIGIT play a function role in Tregs, Joller et al. (134) first identified not only is TIGIT majorly expressed on natural Tregs but also can promote induced Tregs differentiation. Intriguingly, TIGIT marks a functionally distinct subset of human nTregs with superior suppressive activity *in vitro* (134). The inhibitory assay result revealed that TIGIT^+^ Tregs inhibit cell differentiation and responses of Th1 and Th17 subsets but promote Th2 immunity through a fibrinogen-like 2-dependent mechanism (134). In addition, as a potent immunosuppressive molecule, TIGIT penetrate through early, middle, and late stages of the cancer immunity cycle (135). Besides, CD155 and CD112, ligands of TIGIT, are highly expressed on various human tumors besides immune cells (136), which indirectly suggest the involvement of TIGIT in tumor immunity. Taking into account the crucial roles of TIGIT in immunosuppression, and benefits from TIGIT blocking in animal studies or *in vitro* experiments, TIGIT blockade alone or together with other coinhibitory molecules would be considered as a potential therapeutic strategy for tumor management.

Lymphocyte activation gene-3 (CD223) was discovered 25 years ago as an activation marker (137). Due to its highly homologous structure to CD4, LAG-3 also binds to MHC II molecules with a much higher affinity. LAG-3 is not expressed by quiescent T cells but is up-regulated several days after activation (138). In contrast to effector T cells, LAG3 is highly expressed on CD4^+^ T cells that have regulatory functions, including activated natural Tregs, induced Tregs and type 1 regulatory (Tr1) T cells (139). Micro-scaled functional assays showed binding of LAG-3 to MHC II molecules induce an immunoreceptor tyrosine-based activation motif-mediated inhibitory signaling pathway (140). LAG-3 seems essential for maximal Tregs suppression since blockade of LAG-3 on Tregs abrogates their suppressor function (141). But the concrete role of LAG-3 for Treg-mediated suppression remains controversial. Some immunologists speculated maybe LAG-3 was essential for Treg-mediated suppressive activity at high effector T/Treg ratios but being dispensable at lower ratios (142). With regards to Tr1 cells, LAG-3 acts in Tr1 induction and its function indispensably since it is further expressed on Tr1 cells. In addition, coexpression of CD49b and LAG-3 can be utilized to authenticate Tr1 cell lineage in human and mice, solving the ambiguous problem of Tr1 cells identification in a large extent (143). However, opponents argued that inhibitory receptors cannot be supposed to ideal surface marker for Tr1 cells due to their dynamic expression (144). Anyway, the application of LAG-3 in clinical practice seems inexorable. Presently, LAG-3 has been shown to be an important immune regulator in autoimmunity (145), chronic viral infection (146), parasitic infection (147), and cancer (148). Specially, in the setting of advanced cancer, LAG-3 is preferentially expressed on tumor-infiltrating Tregs (149) and these Tregs display a terminal effector phenotype and proliferate less than LAG3^−^ Tregs (150). LAG3-specific monoclonal antibody and LAG-Ig (IMP321) are in early phase clinical trials for cancer, these trials are still recruiting patients and thus it will be some time before trial data are available (151). It is noteworthy that human Tregs can acquire MHC II molecules *via* trogocytosis (152) and human MHC II^+^ Tregs had been proved more suppressive than MHC II^−^ Tregs (153). Considering inextricable relationship between MHC II and LAG-3, we can presume LAG-3 engagement escort stronger suppressive activity of MHC II^+^ Tregs. Considering the potential role of MHC II on Tregs in mediating immune inhibition through LAG-3, interfering with the LAG-3/MHC class II pathway may help to prime or potentiate preexisting T cell responses to tumor antigens. Results of preclinical models also strengthened the rationale to further study of LAG-3.

T-cell immunoglobulin-3 (CD366) was identified in 2002 and alongside TIGIT, LAG-3 represent the next generation of immune checkpoints in cancer immunotherapy. Tim has been defined as a negative regulatory molecule and acts as an indispensable role in immune tolerance. Tim-3 is expressed by Tregs and is also found on other lymphocytes such as effector T cells, NK cells, DCs, and monocytes. Besides galectin-9, phosphatidyl serine, high mobility group protein B1, carcinoembryonic antigen cell adhesion molecule-1 was identified as another novel cell surface ligand of Tim-3 recently (154). The functional regulation of Tim-3 on regulatory T cells is achieved just by binding to these ligands. Specially, Tim-3 is expressed on only a few subsets of CD4^+^Foxp3^+^ Tregs after TCR stimulation but the level is obviously upregulated on them at the sites of tissue inflammation (139). Intriguingly, other coinhibitory elements such as LAG-3, PD-1, and CTLA-4 were also upregulated on TIM-3^+^ Tregs. More importantly, Tim-3^+^ Tregs show superior immuno-suppressive activity when compared to Tim-3^−^ Tregs. They preferentially express higher levels of known suppressive molecules including IL-10, granzymes and perforin (155). Notably, Tim-3^+^Tregs were reported, in particular, suppress Th17 cells while Tim-3^-^Tregs did not (155). In human tumor microenvironment, Tim-3^+^ Tregs form the predominant subpopulation throughout all phases of tumor progression (156). The presence of Tim-3^+^ Treg cells has been found to associate with unfavorable prognostic parameters such as nodal metastases in lung cancer, further supporting the value of Tim-3 as a prognostic indicator of disease progression (157). As mentioned above, either CTLA4 inhibitor or PD-1 inhibitors did not give rise to high response rate in the treatment of patients with some cancer types. It is quite urgent to identify other checkpoint inhibitors to be used in monotherapy or combotherapy with PD-1/PD-L1 inhibitors. In this context, Tim-3 marks highly suppressive Tregs that are present uniquely within the tumor microenvironment. Tim-3-targeted therapy alone or in combination with other checkpoint therapies are emerging as a potential treatment modality for further improvement of current immunotherapy.

## Concluding Remarks

Since their discovery as a key mediator of immunological self-tolerance, considerable progress has been made in Tregs. However, the original recognition of Treg should be revised and updated since plenty of recent work subvert our previous understanding. For example, Tregs are not the most potent suppressive players in some tumor-settings (158, 159). In addition, an emerging body of research suggests that tissue-resident Tregs have specialized functions that are unique to the tissues they reside. It goes without saying that understanding tissue Tregs in diverse regions of the body would yield copious novel immunological principles. The identification of novel tissue-specific Tregs has highlighted their heterogeneity and complexity. We should focus on not only their specialized roles in regulating immune responses but also their tissue-specific functions. Following the updated cognition of Tregs, most of the contents in this Review were discussed in terms of small molecules or functional proteins.

Tregs are capable of suppressing not only conventional T cells but also B cells, NK cells, dendritic cells, and macrophages *via* humoral and cell-cell contact mechanisms (1, 160). An increasing number of molecules are proven to participate in Treg-mediated suppression process, including IL-2, CTLA-4, TGF-β, LAG3, GITR, granzyme B. Since these basic mechanisms have been studied and reviewed extensively, it is not absolutely essential to explore them here in detail. Remarkable progress has been made of late years in expounding the mechanisms that Tregs manipulate to exert suppression activity. But from conclusive, present research just got infinite nearly fact and what we have known remains in its infancy.

In the early stage, efforts focused on ways to use cytokines to manipulate the host immune response toward cancer cell recognition and eradication. Though significant advances were achieved with interleukin-2 and interferon-α, their applications have not been established largely because of toxicity, the complex functionality and the difficulty mimicking human environment. Similarly, further study is required to evaluate the efficacy and safety of IL-34, IL-35, etc. treatment.

Afterward, multiple means to target intratumoral Tregs include small molecules are being conducted in early phase clinical trials, such as TRX518, anti-CCR4, OX40, and GITR. Recently, Neuropilin-1, amphiregulin, etc. constitute a more preferable approach to target Tregs without generating severe side effects.

Substantial data already exist that combinational treatments might be more beneficial than anti-CTLA-4 and anti-PD-1/PD-L1 monotherapies. Considering the complexity of multiple immune checkpoints expression and their ligand, it seems necessary to evaluate combinatorial components within the tumor microenvironment. Presently, technologies using chimeric antigen receptor-engineered T cells is booming with high expectations for cancer immunotherapy, and Kymriah has been approved by FDA to treat B cell acute lymphoblastic leukemia. It also has applications for engineering antigen-specific Tregs to combat neoplastic diseases.

Tregs are attractive targets for immunotherapy, but a better understanding of population dynamics, and the diversity of subphenotypes is worthy of substantial additional investigation. Given the complexity of Treg biology in human diseases, targeting Treg immune-suppressing pathways for the prevention and/or treatment of human diseases requires careful evaluation on the nature of immune response in human diseases. It is said “despite twists and turns on the road ahead, there are bright prospects for this cause.”

## Author Contributions

Each author has participated sufficiently in the work to take public responsibility for appropriate portions of the content.

## Conflict of Interest Statement

The authors are not aware of any affiliations, memberships, funding, or financial holdings that might be perceived as affecting the objectivity of this review.
